# Ronald Grey Gordon

**Published:** 1950-07

**Authors:** 


### Obituary

---

### RONALD GREY GORDON

M.D., D.Sc. (Edinburgh); F.R.C.P. (Edinburgh)

The sudden death of Dr. R. G. Gordon at Bath on April 26th has deprived us of one of our best-known physicians. Dr. Gordon was educated at Charterhouse and at Edinburgh. After qualifying, he held house appointments there and at the Paddington Green Children's Hospital, and came to Bath shortly before the 1914 war: he served four years in the R.A.M.C., at Salonika and in France. Soon after his return to Bath, he was elected to the Royal Mineral Water Hospital, later the Royal National Hospital for Rheumatic Disease. His main interests were neurology and hydrotherapy, and he was on the staffs of several hospitals—the Bath Royal United, Bath Orthopaedic the Ministry of Pensions Hospital at Bath, and Stoke Park Colony.

He was elected F.R.C.P. (Edinburgh) in 1926 and was Morison Lecturer in 1947: he was a Fellow of the Royal Society of Medicine, Fellow and ex President of the Royal Medical Society of Edinburgh, twice Chairman of the Bath Division, and in 1947, President of the Bath, Bristol and Somerset Branch of the B.M.A. In 1929 he was elected to the Council of the B.M.A. and shortly after this became Chairman of the *Journal* Committee: he took a keen interest in the details of *Journal* production and was largely responsible for the reorganization and new format introduced twelve years ago. In addition to sections of several books on neurology and psychiatry he wrote *The Neurotic Personality* and *Autolycus: the Future for Miscreant Youth.* As though all this were not enough, with a very busy practice, he served on numerous committees for the subjects he had most at heart and was noted for his enthusiasm and practical contributions. A truly great physician.

To his family we tender our sincere sympathies.

